# Numerical Reliability Study Based on Rheological Input for Bingham Paste Pumping Using a Finite Volume Approach in OpenFOAM

**DOI:** 10.3390/ma14175011

**Published:** 2021-09-02

**Authors:** Robin De Schryver, Khadija El Cheikh, Karel Lesage, Mert Yücel Yardimci, Geert De Schutter

**Affiliations:** Magnel-Vandepitte Laboratory, Department of Structural Engineering and Building Materials, Faculty of Engineering and Architecture, Ghent University, Technologiepark-Zwijnaarde 60, B-9052 Ghent, Belgium; Robin.DeSchryver@UGent.be (R.D.S.); Khadija.El.Cheikh@bbri.be (K.E.C.); Karel.Lesage@UGent.be (K.L.); MertYucel.Yardimci@UGent.be (M.Y.Y.)

**Keywords:** numerical simulation, computational fluid dynamics, OpenFOAM, cementitious materials, Bingham rheology, pumping, reliability

## Abstract

Rheological quantification is important in many industries, the concrete industry in particular, e.g., pumping, form filling, etc. Instead of performing expensive and time-consuming experiments, numerical simulations are a powerful means in view of rheological assessment. However, due to the unclear numerical reliability and the uncertainty of rheological input data, it is important for the construction industry to assess the numerical outcome. To reduce the numerical domain of cementitious suspensions, we assessed the numerical finite volume simulations of Bingham paste pumping flows in OpenFOAM. We analysed the numerical reliability, first, irrespective of its rheological input by comparison with the literature and theory, and second, dependent on a certain rheological quantification by comparison with pumping experiments. Irrespective of the rheological input, the numerical results were significantly accurate. Dependent on the rheological input, a numerical mismatch, however, existed. Errors below 1% can be expected for proposed numerical rules of thumb: a bi-viscous regularisation, with pressure numbers higher than 5/4. To improve bias due to uncertain rheology, a rheological configuration close to the engineer’s aimed application should be used. However, important phenomena should not be overlooked. Further assessment for lubrication flows, in, e.g., concrete pumping, is still necessary to address concerns of reliability and stability.

## 1. Introduction

Concrete structures and their finishing quality, durability or even structural integrity passively rely on the concrete’s fresh state properties [1], more specifically, the fresh state flow characteristics, which are usually characterised by so-called rheological parameters. The rheology of concrete and more general cementitious-like suspensions, which are mostly governed by Bingham flow behaviour, therefore determines the outcome of its application [1]. For simple and, more generally, industrial processes, one could predict the outcome based on existing theories. For more complicated processes, this is not the case. As a first alternative, the outcome of an industrial process could then be assessed based on experiments. Experimental tests can, however, be inconvenient, time consuming and/or expensive. As a second alternative, one could therefore use numerical simulations to overcome this issue.

The numerical simulation of cementitious suspensions, Bingham flows in general, shows a huge potential for predicting the flow behaviour in several construction or industrial processes. Numerical simulations for predicting industrial (construction) processes could for instance provide the required pumping pressure, form filling ability, etc. One could even use numerical simulations to predict more complicated behavioural aspects such as particle migration or time-dependent behaviour such as thixotropy, which can be a major cause for blocking problems [2,3,4,5,6,7,8]. Moreover, one could use numerical simulations for industrial design.

One popular approach to numerically simulate cementitious suspensions or Bingham flows in general is computational fluid dynamics (CFD) [3,5,6,8,9,10,11,12]. CFD considers a homogenous fluid that can be justified to simulate flows of cementitious materials [3,6]. Since a continuous approach is involved in CFD, particle behaviour aspects may only be considered from a macroscopic point of view [3,5]. If one is interested in specific particle phenomena occurring in, e.g., concrete flows, other techniques such as discrete element method (DEM) and lattice Boltzmann method (LBM) are advised [5]. The advantage of CFD over other techniques is that it allows for faster computations and larger computation domains, which are suitable for practical simulations such as pumping, formwork filling, on-site rheological test cases, etc. [6,8,13]. The amount of numerical mesh cells is still considerable (ca. $10^{4}$–$10^{6}$ cells [3,5,6,8,9,13]) without the requirement of special computational infrastructure. Therefore, CFD could serve as a useful tool within practical reach for design engineers. Many commercial CFD softwares on the market, however, are bounded by licences that can be expensive. Therefore, open-source CFD codes have been rising in popularity because they are accessible for anyone. Instead of a black box-embedded CFD code, the source code is accessible and modifiable for any prerequisite needed. One open-source software in particular, named OpenFOAM, has been rising in popularity over the past decades and is therefore convenient or suitable for use in applications of the modern industries, particularly among the concrete industry. Since numerical simulations use rheological data for their input values, the numerical simulations themselves are an immediate consequence of the rheological device or characterisation procedure. Several devices, known as rheometers, exist to quantify rheological data for cementitious suspensions together with their respective geometries, such as ConTech Viscometer, Tattersall MK-II, ICAR, Anton Paar MCR device, Sliper and more [14,15,16,17]. Despite the numerous existence of rheological devices, the rheological parameters obtained by these devices differ [14,15,16,17,18,19]. Worse is that the difference among devices can be significant [14,15,19,20,21].

Irrespective of the rheological input, some works assessing the numerical accuracy for Bingham flows exist [21,22,23,24,25,26,27,28,29]. However, these studies are in terms of error in numerical residuals or numerical stability criteria with regard to numerical regularisation techniques. Therefore, they may somewhat lack genuine interpretation of the actual numerical outcome or reliability. In the context of cementitious materials, some numerical studies have also been conducted to numerically simulate cementitious paste suspensions or even concrete suspensions [2,3,5,6,7,8,9,13,30,31,32,33,34,35]. However, in most of these works, it is unclear how reliable the numerical simulations actually are, independent of whether a mismatch or good match was found, let alone to know the actual influence of the rheological input with regard to the numerical outcome. Therefore, the question remains how reliable are numerical simulations based on their rheological input and how can more reliable results be obtained, especially because of the disparity in rheological data? Moreover, some devices are reliable for only certain rheological properties and/or types of concretes or suspensions, e.g., paste, traditional concrete and self-compacting concrete. Since numerical simulations rely on rheological input values and therefore on their uncertain character, it is important to assess how reliable the numerical simulations are based on their input. In that way, confidence can be built in the simulation of Bingham suspension flows and, by extension, for engineers practicing in the industry. One example, in particular, is to model fresh concrete construction processes in the concrete industry. The uncertainty problem is twofold: on the one hand, the uncertain character of the rheological input data and, on the other, the accuracy of the numerical outcome itself.

**Figure 1:**
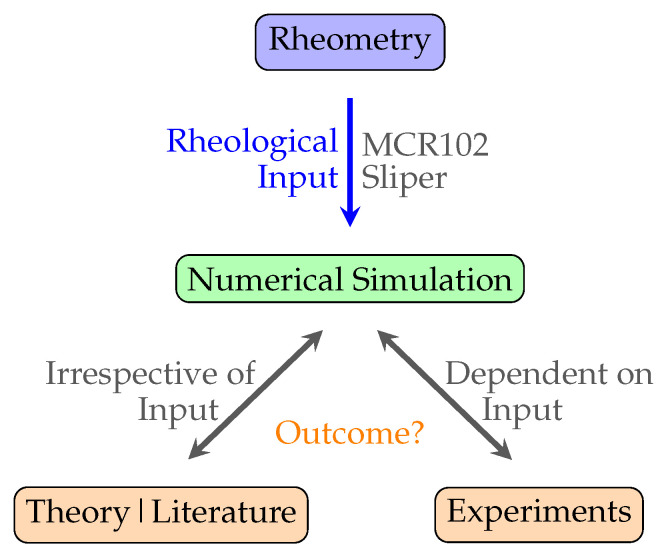
Reliability overview of the input for numerical simulations.

To simplify the vast numerical uncertainty problem in this study, we assessed the accuracy of the numerical simulations for the simple case of pumping of Bingham pastes in the context of cementitious paste suspensions. A potential follow-up in the context of the concrete industry is the more complicated concrete pumping flows, in which an additional pipe wall lubrication layer is expected to contribute to the flow. This follow-up has not been undertaken to not further complicate the conciseness and findings of this work. In that way, we provide a validation framework for engineers in practice to model laminar Bingham flows; simple cementitious suspensions; or perhaps, by extension, even projections for fresh concrete construction processes. To deal with the twofold character of the uncertainty problem, we decomposed it into a first part by assessing numerical simulations using the expected theory or literature and a second part by assessing the simulations based on experiments. The twofold problem decomposition is graphically depicted in Figure 1. Hence, the first decomposed part is irrespective of its rheological input, while the second decomposed part depends on a certain rheological input.

To do so, we first elaborate upon the numerical methodology used, which is a CFD finite volume approach in OpenFOAM to simulate horizontal, laminar pipe flow of cementitious-like Bingham pastes. The numerical framework is followed by the experimental framework for all performed tests. Then, the results for the first and respective second problem decomposition parts are outlined. In the first part, numerical simulations are compared to expected literature results and theories. In the second part, simulations are compared to experimental pumping observations based on a certain rheological input. In that way, we are able to assess the numerical outcome irrespective of the rheological input as well as the reliability based on certain rheological data. Finally, the results are discussed and concluded.

## 2. Numerical Framework

The popular open-source software OpenFOAM (v5, The OpenFOAM Foundation Ltd., London, UK) is used in this framework to assess the numerical adequacy of modelling Bingham cementitious-like suspension pipe flows. We therefore outline the numerical methodology, regularisation, control, case set-up and mesh independence.

### 2.1. Numerical Methodology

Proper to CFD, OpenFOAM models the fluid domain as a continuum with macroscopic properties [3,8]. Doing so, the numerical solution is in fact an approximation of reality constrained by its discretisation (mesh), solving algorithm (solver), solver settings and imposed assumptions. The numerical solution is obtained by solving the fluid continuum’s conservation laws of mass and momentum. This results in solving the continuum Equation (1) and the Navier–Stokes Equation (2) because cementitious-like material flows are incrompressible with a constant density ρ [3,8,36].

(1) $$\begin{array}{cc} \nabla \;\cdot \;U & =0 \end{array}$$

(2) $$\begin{array}{cc} \rho \frac{DU}{Dt} & =\rho g-\nabla p+\nabla \;\cdot \;\left(\nu \nabla U\right) \end{array}$$

In these equations, *U* is the velocity vector field and *p* the pressure scalar field. In the used solver, gravity *g* is omitted because gravity is invariant in the case of horizontal pipe flow, as considered in this paper. Therefore, the pressure *p* and viscosity ν are computed in kinematic form, which is relative to the density ρ. The material derivative is denoted by $\frac{DX}{Dt}=\frac{\partial X}{\partial t}+U\;\cdot \;\nabla X$, in which · is the inner (tensor) product.

Cement pastes exhibit non-Newtonian flow behaviour. By omitting time-dependent behaviour, they are usually modelled as a Bingham fluid with a yield stress $\tau_{0}$ and a plastic viscosity μ[10,37,38,39,40,41,42]. The non-Newtonian character is therefore numerically coped with by using a Generalised Newtonian Fluid (GNF) approach in which the Newtonian viscosity ν is replaced by an apparent viscosity η computed for each mesh cell corresponding to the considered constitutive rheological model, i.e., the Bingham model. In doing so, a numerical singularity occurs for zones with zero or low shear rates (i.e., quiescent zones). Eventually, this can lead to numerical stability issues. This is the so-called unregularised viscoplastic problem [43]. In the literature, this problem is either coped with by making use of a regularisation approach or a different modelling technique (e.g., augmented Langrangian method) [25,43]. Other examples of numerical techniques where this problem is coped with were conducted by, e.g., Jahromi et al. [28], Bleyer et al. [44], Pimenta and Alves [45]. Using such advanced numerical techniques is however not straightforward. Therefore, the bi-linear regularisation approach is used in this work.

### 2.2. Numerical Regularisation

Several regularisation approaches have been investigated in the literature such as the bi-linear [23,24,46], Bercovier and Engelman [22], Papanastasiou [10,12,47,48,49] and other approaches [25,43,50]. These regularisation approaches restrain the infinite apparent viscosity η for zero shear rates, usually defined by a so-called epsilon ε environment. The smaller ε, the higher the apparent viscosity can reach, resulting in an overall more stiff or viscous flow behaviour in quiescent zones [43]. Higher apparent viscosities lead to better approximations of the non-Newtonian yield character; it may however also inflict numerical stability issues [43].

For ease of implementation and interpretation, the bi-linear regularisation approach [23] is used in this work by restraining the apparent viscosity to a maximum value, similar to [21]. A constant Generalised Newtonian Approach Ratio (GNAR) is therefore defined. This is the ratio of the maximum apparent viscosity $\eta_{max}$ and plastic viscosity μ because it defines the order of magnitude and therefore the dominance of the numerical matrices in which the apparent viscosity η is used. This approach of limiting the ratio to 1000 (or an equivalent regularisation parameter) is in line with the philosophy by Schaer et al. [21], Bercovier and Engelman [22], Ahmadi and Karimfazli [29], Papanastasiou [47]. Moreover, this is exactly the same approach and recommendations as Beverly and Tanner [24], Bullough et al. [46] and Burgos et al. [51]. Thus, the higher the GNAR, the better the non-Newtonian character but the more the numerical simulation is subjected to stability issues, especially in quiescent zones. Quiescent zones are zones with very low or zero shear rates, such as the central plug zone in pipe flows. Based on a parametric sensitivity study, we used a GNAR of 1000 to obtain better yield behaviour predictions.

### 2.3. Numerical Control

To additionally cope with numerical instability problems for simulations with big plug zones (quiescent zones), under-relaxation of the velocity field *U* was used to a limited extent of 0.95. This could be considered a similar but more convenient and easy to implement relaxation approach as used by Chupin and Dubois [52]. Since under-relaxation smears out the numerical solution over time or iterations, we advise limiting the velocity under-relaxation between $\left[0.95,1.0\right]$. This is based on a time-dependent parameter study of the under-relaxation. This helped us to overcome all numerical GNF instability issues while still respecting the temporal flow behaviour to a reasonable extent.

In order to ascertain temporal numerical stability, the well-known CFL (Courant–Friedrichs–Lewy) number was controlled and limited to 0.5. We gradually increased the CFL number after flow initiation from 0.001 to 0.5 to allow for faster numerical progress because no temporal boundary conditions were considered and hence the initiation phase is an immediate flow jump, starting from a zero velocity to a velocity corresponding to the imposed discharge *Q*. Hence, the initiation may lead to initial numerical instability issues, which have been coped with by gradually increasing the CFL number.

The total simulation time was 60 s, performed by the transient (PISO: Pressure-Implicit with Splitting of Operators) OpenFOAM solver called nonNewtonianIcoFoam, adopted for a customly developed Bingham model. The numerical data were analysed using a Python script, considering steady-state only for the data analysis.

### 2.4. Numerical Cases

Several numerical pipe flow simulations were performed in order to assess their reliability irrespective of the rheological input. On the one hand, 10 pipe flow simulations were performed to compare the results with the literature results from Tichko [13] for a DN100 (106 mm) pipe. On the other hand, to compare simulation results with the Buckingham–Reiner theory, 213 simulations were additionally conducted, re-simulating small-scale pumping experiments that were performed with a DN25 (26.64 mm) pipeline. Simultaneously, these 213 pipe flow simulations served to assess the reliability dependent on the rheological input by comparing the numerical results with the experimental observations. Hence, a total of 223 numerical cases were considered, disregarding the 660 numerical cases conducted for sensitivity studies.

The boundary conditions (BC) are also important, since they determine how a simulation domain interacts with the physical outer world. Therefore, the pipe boundary conditions are depicted in Table 1.

A default set of numerical solvers and schemes (mostly linear) are used for the considered simulations. Even though higher-order schemes and better-performing solvers would facilitate the numerical accuracy—though it has an additional computational cost—it is not the aim of this work to outline the most optimal settings. Our aim is rather to give a global idea on the accuracy, be it with rather default-like or CFD-advised settings. Further details on the numerical schemes and settings can be consulted in the respective Appendix A. In that way, a benchmark may be obtained for simulation engineers in practice or in related research domains.

**Figure 2:**
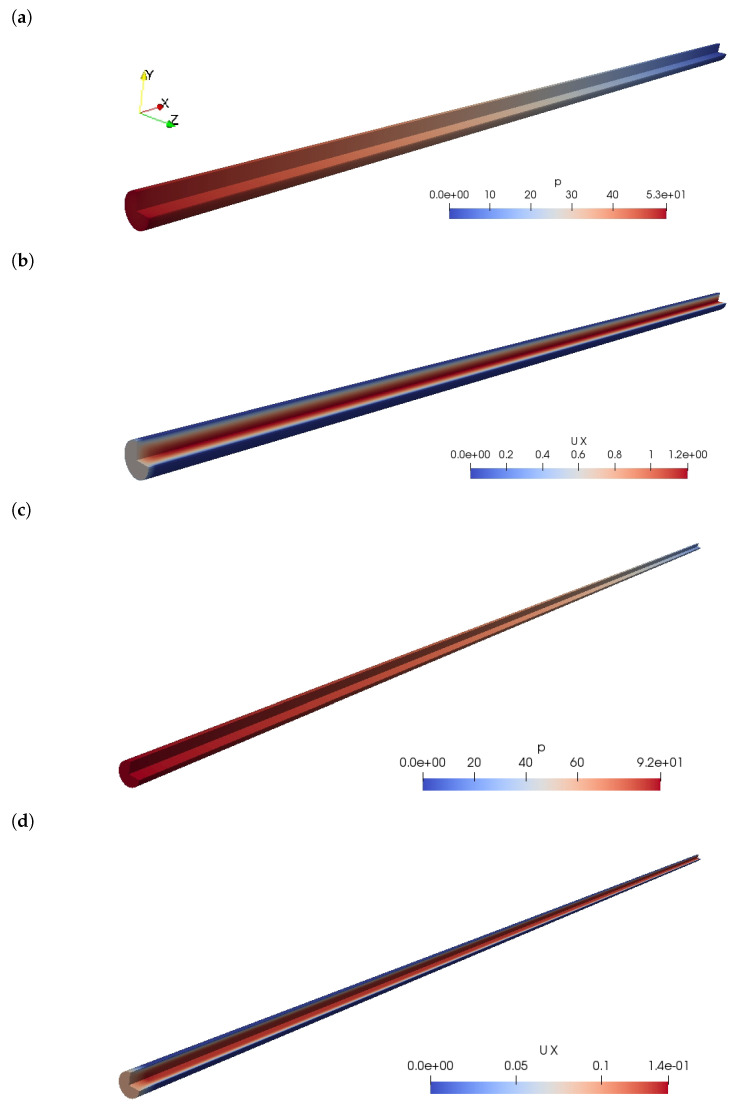
Illustrations of the numerical outcome. Examples of the kinematic pressure field *p* (m${}^{2}$/s${}^{2}$) and the x-component velocity field $U_{x}$ (m/s) are shown for both the DN100 and DN25 pipe cases. (**a**) Pressure field *p* for a DN100 pipe. (**b**) Velocity field $U_{x}$ for a DN100 pipe. (**c**) Pressure field *p* for a DN25 pipe. (**d**) Velocity field $U_{x}$ for a DN25 pipe.

Examples of the typical numerical outcome of the pipe flow simulations are illustrated in Figure 2. For every simulation, the 3D pressure and velocity field are simulated, after which the fully developed pressure loss is processed for further analysis in this work. For steady-state, these illustrations show a linear pressure loss as well as a quadratic velocity profile with central plug flow. This is expected from laminar Bingham simulations [53].

### 2.5. Mesh Independence

**Figure 3:**
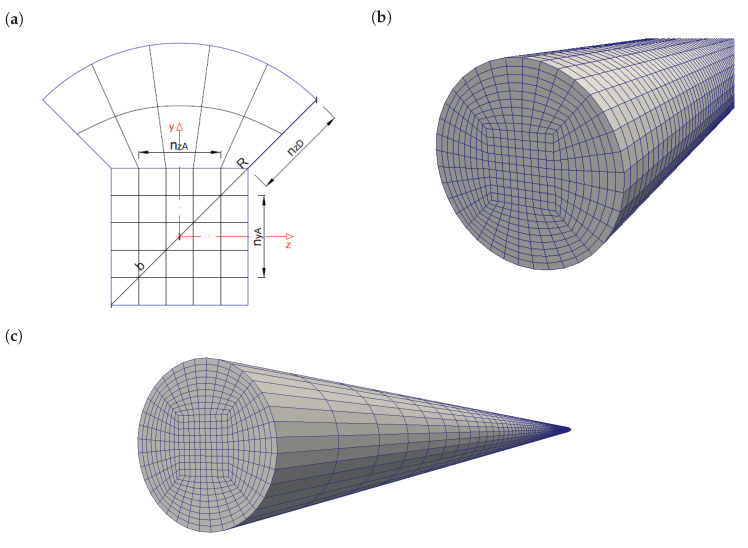
Hexagonal cross-sectional mesh configuration, with an inner square ($n_{yA}$×$n_{zA}$) with diagonal 2b and outer circle segments ($n_{zA}$×$n_{zD}$) to pipe radius *R*. (**a**) Cross-sectional view. (**b**) Three-dimensionally rendered mesh view of a DN100 pipe. (**c**) Rescaled mesh of experimental pumping circuit, with an equal radial mesh resolution for a DN25 pipe.

A last aspect that is to be considered for a simulation is the mesh quality. The mesh quality has a big influence on the obtained results in numerical modelling studies. Therefore, the simulations should be mesh independent. A mesh independence study was performed for pipe flow simulations. The acceptance criteria for mesh independence were based on mesh orthogonality; mesh angularity; a mesh verification check by OpenFOAM; and more importantly, convergence of simulated pressure loss results, indicating true independence. To respect orthogonality, a cylindrical hexagonal mesh configuration of the pipe was used as depicted in Figure 3. Based on the independence analysis, the final cross-sectional mesh configuration consisted of 9×9 inner square elements (to respect orthogonality) and compatible 9×9 outer circular section elements (on the four sides). The inner square diagonal to pipe diameter ratio was 0.5 (Figure 3a). The longitudinal mesh was uniformly subdivided into elements with a length of 2 cm.

The mesh independence analysis was based on a pipe with a diameter of 106 mm (DN100), which was used to compare the simulations with literature. Since the pipe diameter of the conducted pumping experiments is smaller, the radial configuration is scaled down by maintaining the same mesh numbers (Figure 3c). Longitudinally, it is assumed to be sufficiently refined, and a mesh cell length of 2 cm was not further refined. This mesh scaling is justified since the flow can radially develop over the same amount of mesh cells. Longitudinally, it is also justified because the simulated pressure losses used in the analysis are verified fully developed pressure losses. Moreover, this re-scaling is even proven to be justified by the fact that the simulation results of DN100 pipes have the same relative theoretical error (ca. 1%) as DN25 pipes, as outlined in Section 4.1 and Section 4.2 respectively.

## 3. Experimental Framework

The experimental work consisted of two stages. First, a rheological characterisation was performed for all considered samples by rheometer tests. Second, pumping experiments were performed to assess the reliability of the numerical simulations based on rheological input data. In total, four different paste mixture designs (A1 to A4 in Table 2) were tested at least three times to ensure the repeatability.

The pastes used in the experiments were in fact Bingham model fluid pastes, without thixotropic and hydration-related properties, to increase the experimental time window, instead of actual cement pastes. The model pastes consisted of limestone powder (P; with a median particle size of 3.7 μm), water (W) and/or a polycarboxylate ether-based superplasticiser (SP). The four different paste designs represent a rheological range of cement pastes with a low/high yield stress $\tau_{0}$ and a low/high plastic viscosity μ. Hence, the terminology of cementitious-like pastes is preferred, since the model fluids represent typical cement pastes.

### 3.1. Rheometry

The Bingham behaviour of the model pastes were rheologically characterised by two approaches. The first approach is a characterisation based on a flow curve protocol in a rotational rheometer (MCR-102, Anton Paar Benelux BVBA, Gentbrugge, Belgium). The second rheological characterisation is based on a sliding pipe rheometer (Sliper, Schleibinger Geräte Teubert und Greim GmbH, Buchbach, Germany) [54].

#### 3.1.1. Rotational Rheometry

**Figure 4:**
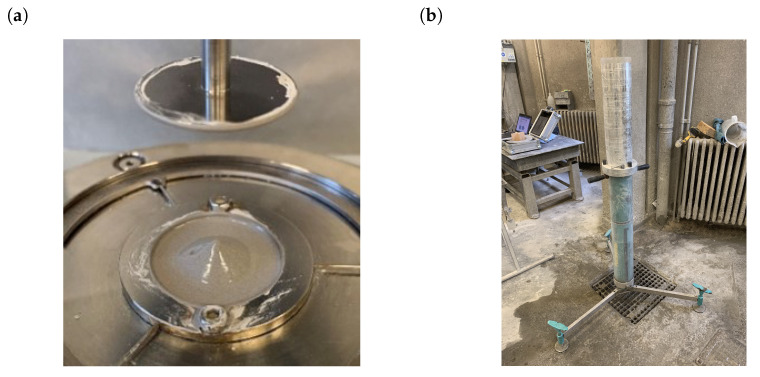
Rheometer test set-up configurations: (**a**) rotational parallel plate rheometer configuration (Anton Paar MCR-102 with a 1 mm gap); (**b**) sliding pipe rheometer (Sliper).

For all paste samples, rheological tests were performed with a parallel plate configuration in a rotational rheometer (MCR-102, Anton Paar Benelux BVBA, Gentbrugge, Belgium). A constant temperature of ca. 20 ${}^{\circ }$C temperature was maintained during the testing procedure by the rheometer’s temperature control unit. The considered parallel plate geometry (1 mm gap) is shown in Figure 4.

The testing procedure consisted of a flow curve test anticipated by a pre-shear (20 s at 120 rad/s). The flow curve test consisted of a shear step-up and step-down protocol in which each shear step had a constant shear duration of 20s after an increment or decrement of 20 rad/s. Steady-state values of the step-down parts were used to compute the flow curves.

#### 3.1.2. Sliding Pipe Rheometry

**Figure 5:**
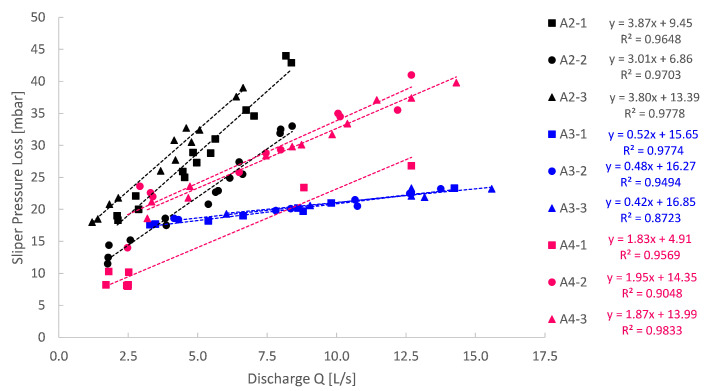
Sliper pressure loss recorded as a function of the discharge for all conducted experiments (mix design A2 to A4). No feasible results could be obtained for mix design A1. Rheological parameters were obtained from the linear regressions.

In addition to the MCR-102 rheometer tests, sliding pipe rheometer (Sliper, Schleibinger Geräte Teubert und Greim GmbH, Buchbach, Germany) tests were also performed for all samples. The Sliper test consists of a pipe (*D* = 125 mm) filled with paste that slides down a platform with a centrally positioned pressure sensor. During this downward motion over a pipe length of 0.5 m, a laser distance sensor captures the velocity of the sliding pipe and thus the discharge of the flow. The geometrical layout of the Sliper is depicted in Figure 4. This sliding procedure is repeated several times each with a different weight attached to the Sliper, allowing for variation in the imposed discharge. In that way, a pressure loss–discharge diagram can be recorded as depicted in Figure 5.

These pressure loss and discharge values were correlated by a linear regression. The linear regressions were then used to characterise the rheological parameters for each of the model pastes based on a reversed engineered Buckingham–Reiner approach. In that way, the Bingham yield stress and plastic viscosity could be obtained by a second rheometric approach. Some Sliper measurements were jeopardised due to bad sensor connectivity. Due to severe leakage, no Sliper measurements could be obtained for mix design A1.

An overview of rheological parameters (i.e., density ρ, Bingham yield stress $\tau_{0}$ and plastic viscosity μ) obtained by both the MCR-102 rheometer and Sliper tests is depicted in Table 3.

### 3.2. Small-Scale Pumping

**Figure 6:**
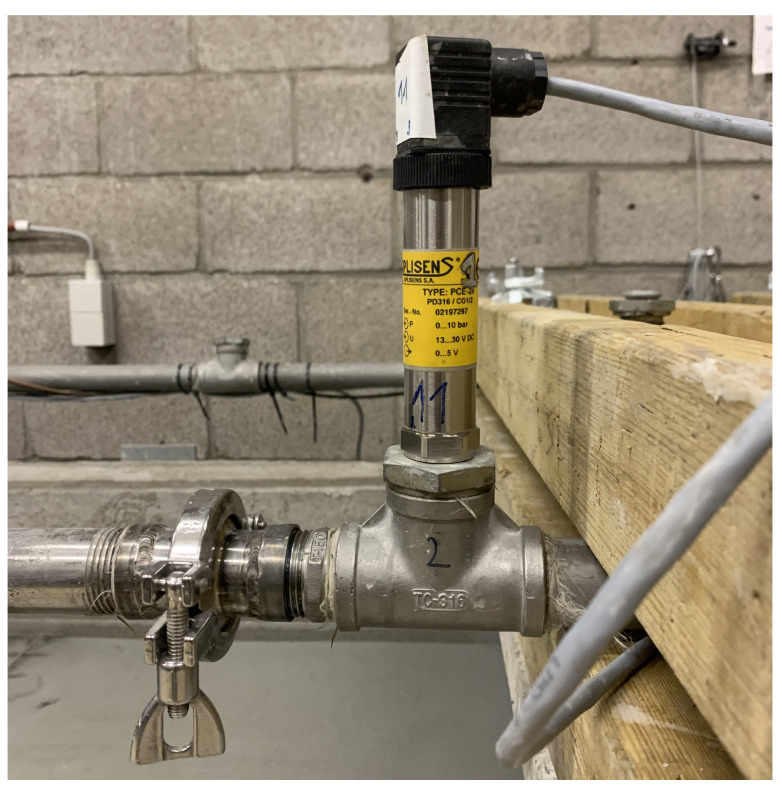
Example of a pressure sensor being screwed in a T-connector as close as possible to the virtual pipe wall.

**Figure 7:**
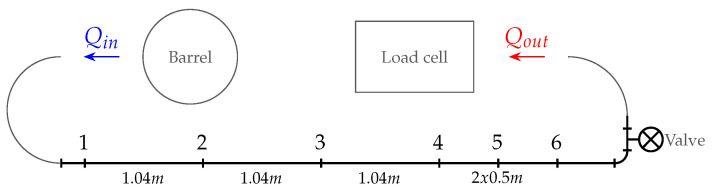
Layout of a small-scale pumping circuit. Each number corresponds to a pressure sensor position. At the inlet, the paste was injected via a pressure barrel, for which the outlet discharge $Q_{out}$ was measured by a load cell.

To quantitatively assess the numerical performance, a validation experiment was set up based on a pumping experiment. A pumping circuit was designed for stainless steel pipes, with an internal diameter of D=26.64 mm (DN25). Several pressure sensors were inserted in the pipeline using T-connections in which the pressure sensors were screwed as close as possible to the (virtual) pipe wall (see Figure 6). In that way, the pressure loss was recorded over respective distances (between pressure sensor 2 and 4 in Figure 7). To also capture the induced discharge, a container was supported from a load cell. The paste was injected into the pumping circuit by a pressure controlled barrel, allowing for a variation in driving pressure and discharge, which were recorded accordingly. The geometric configuration of the small scale pumping set-up (SPS) is depicted in Figure 7. In that way, an experimental validation data set was provided by a pumping experiment.

## 4. Reliability Irrespective of Rheological Input

To assess the reliability of the numerical outcome irrespective of the rheological input, two comparisons were made. First, pipe flow simulations were compared to simulations performed in the literature. Second, simulations were compared with the expected Buckingham–Reiner theory of Bingham Poiseuille flows. This was performed for re-simulated cases of performed small-scale pumping experiments.

### 4.1. Comparison with the Literature

**Figure 8:**
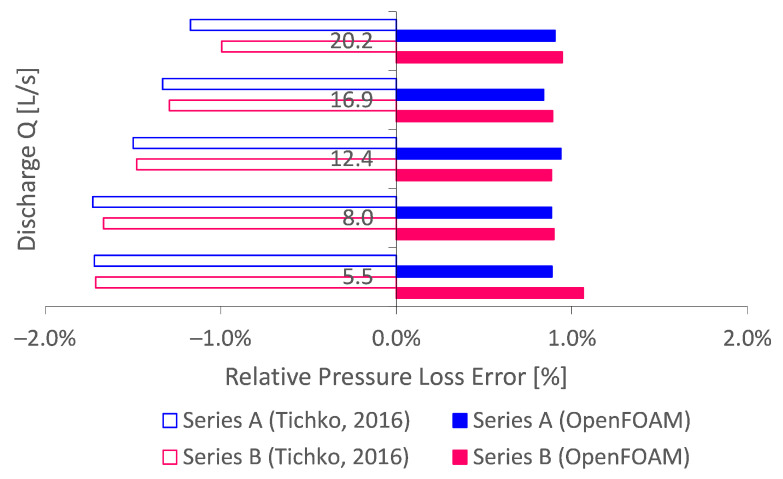
Literature comparison of the numerical concrete pipe flow simulations performed by Tichko [13], relative to the expected Buckingham–Reiner theory. The results indicate that OpenFOAM performs at least as good as, if not better than, commercial CFD software ANSYS FLUENT^®^.

To compare the numerical pipe flow simulations with the literature, pumping simulations of concrete suspensions are compared in particular. This is because cementitious paste suspensions are the main scope of this work. More specifically, we compared the simulations performed by Tichko [13]. Tichko [13] simulated the flow of concrete pumping experiments using a homogeneous suspension approach. Two pumping series A and B were considered in a DN100 pipeline. The lack of wall lubrication layer effect in these simulations is justified from a pure comparison point of view. Figure 8 outlines the comparison of concrete pipe flow simulations by Tichko [13] performed in ANSYS FLUENT^®^, with the re-simulated ones performed in this work by OpenFOAM. No under-relaxation was considered, and a more strict regularisation was applied (GNAR≈1500). The error is presented relative to the expected Buckingham–Reiner pressure loss.

Comparing several discharges, it can be seen that the results in this framework are more accurate with a relative pressure loss error below 1%. Hence, it is concluded that homogeneous Bingham pipe flow simulations performed by OpenFOAM are at least as accurate as, if not better than, simulations by commercial software ANSYS FLUENT^®^.

### 4.2. Comparison with the Theory

To assess the numerical simulations irrespective of their rheological input, they are compared with the expected theory of Buckingham–Reiner. This is based on re-simulated cases of all performed small-scale experiments, as depicted in Table 3.

**Figure 9:**
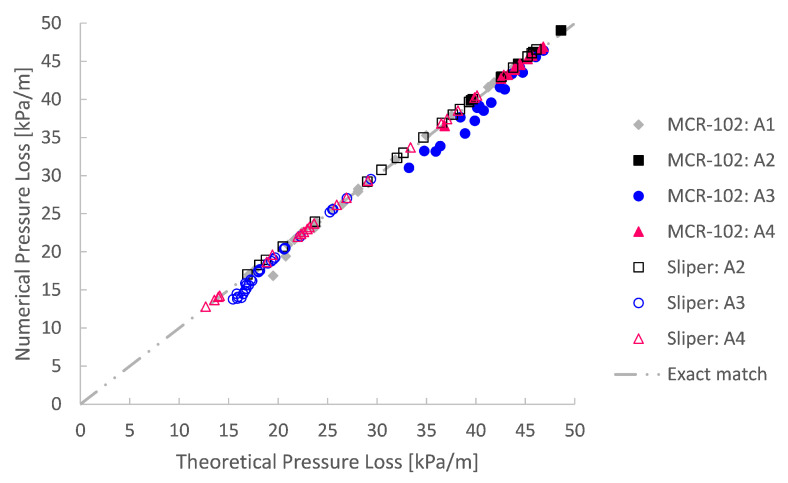
A good agreement is obtained between the simulated pressure loss and the expected theoretical Buckingham–Reiner theory.

**Figure 10:**
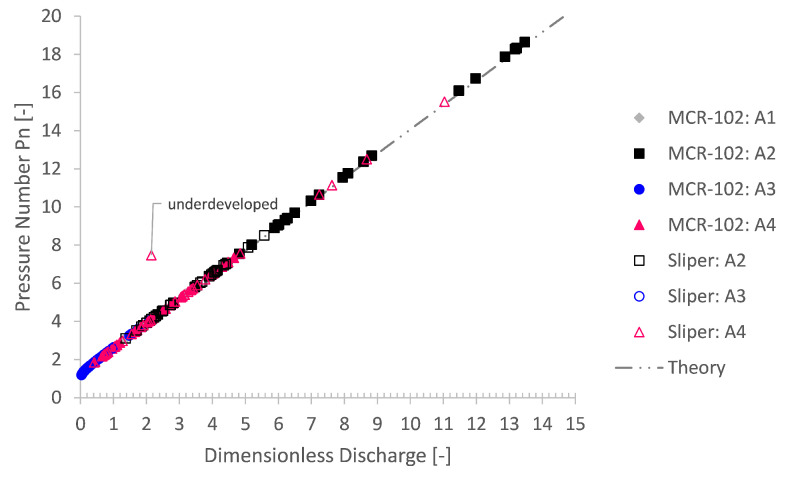
Using a dimensionless form of the Bingham Poiseuille flow, again, a good agreement is achieved between the simulated pressure loss and the expected Buckingham–Reiner theory. Indeed, the simulations map onto a single Bingham discharge curve.

All small-scale pumping experiments were simulated based on their respective rheological input values. To assess these simulations irrespective of their input, they were analysed in comparison with the expected theory of Buckingham–Reiner. This totals to 213 simulation cases. An overview of the simulation outcome is depicted in Figure 9 and Figure 10. Here, the simulated pressure loss is plotted as a function of the discharge. Additionally, the pressure loss as well as the discharge are transformed into dimensionless form via the pressure number $Pn=\Delta pR/(2L\tau_{0})$ and dimensionless discharge $\hat{Q}=Q/\left(R^{3}\tau_{0}/\mu \right)$ after De Schryver and De Schutter [55].

It is clear that the numerical simulations, irrespective of their rheological input, are in good agreement with the Buckingham–Reiner theory (cf. Figure 9 and Figure 10). Indeed, apart from one underdeveloped case, all simulation results map onto a single discharge diagram curve, as expected for pumping of a Bingham fluid [55,56].

**Figure 11:**
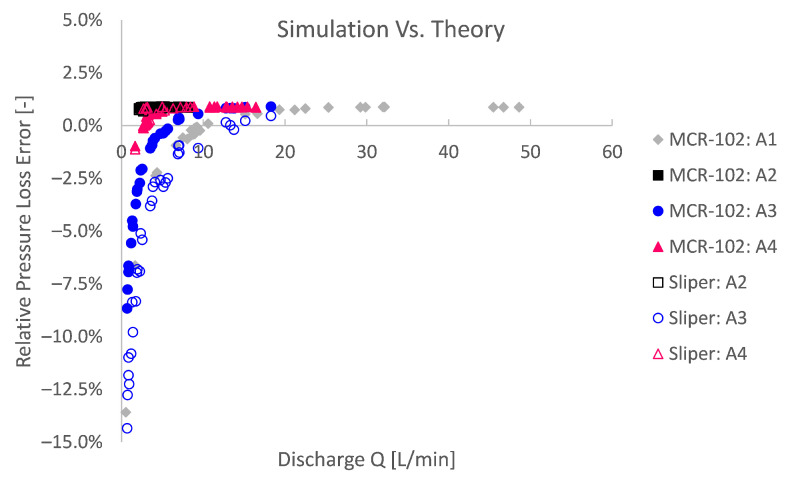
Significantly accurate simulations can be obtained in comparison with the Buckingham–Reiner theory. For higher discharges, a relative pressure loss error below 1% can be obtained. Simulations with lower discharges or higher yield stresses can be more influenced by the unregularised viscoplastic problem [25,43].

To quantitatively assess the agreement, a relative pressure loss error is defined in Figure 11 by comparing the pressure loss from the simulation, with the pressure loss expected by Buckingham–Reiner. The results in Figure 11 show that, for higher discharges *Q*, the simulations are significantly accurate with a relative error below 1%. The convergence to a relative error of 1% for higher discharges may be restrained by the mesh resolution and default-like solving settings. Therefore, it would be expected that, for a proportionally finer mesh resolution as well as higher-order numerical schemes and more restricted solver tolerances, the relative error can be reduced even more. Hence, it is concluded that, if one considers pipe flow with a mesh resolution and default-like solver settings, as considered in this work, no error higher than 1% would be expected. Given this result, one may have confidence in the numerical simulation.

For lower discharges on the other hand, the relative error increases. Since it concerns a relative error, this is partially explained by the lower reference pressure in the division of the relative error. The relative error is namely the absolute error divided by the expected pressure loss, which in its turn decreases for lower discharges.

**Figure 12:**
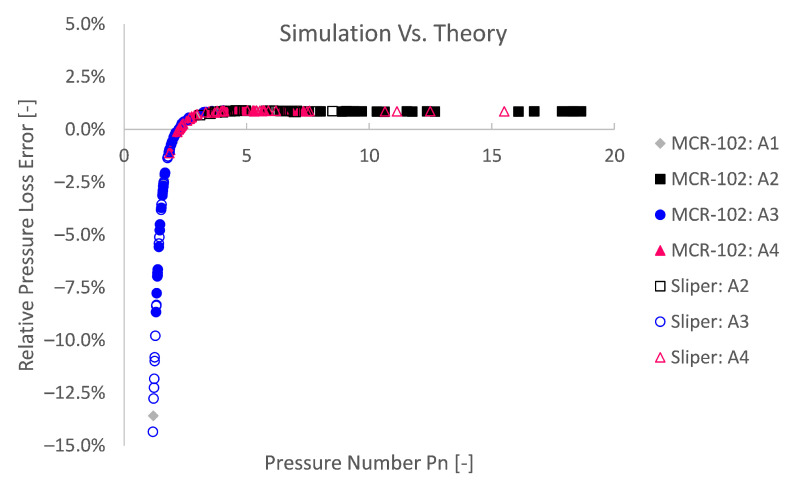
The relative simulation pressure loss error (compared to Buckingham–Reiner) as a function of the pressure number Pn reveals the influence of the flow regime or unregularised viscoplastic problem. The accuracy decreases for lower pressure numbers or for more yield stress dominant flow regimes.

**Figure 13:**
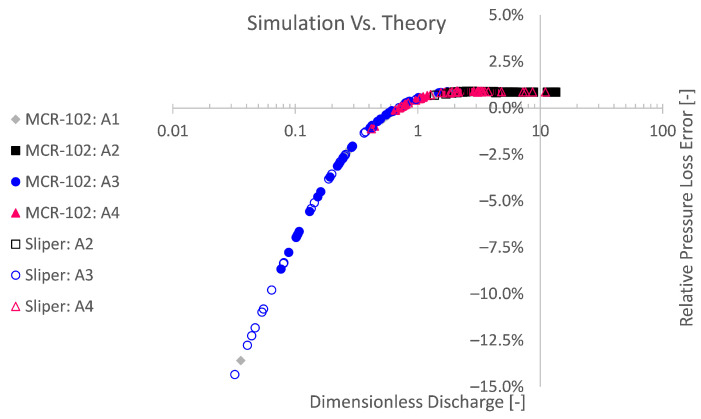
Plotting the relative simulation error as a function of the dimensionless discharge $\hat{Q}$ also shows that low discharges or more yield stress dominated pipe flows impede numerical accuracy.

A second explanation for higher relative theoretical errors is related to the numerical methodology. The Bingham model is approximated by a bi-viscous regularisation approach, in which an apparent viscosity is used, restrained by a pre-defined maximum. On the one hand, this has to be performed to avoid numerical instability. On the other hand, this impedes numerical accuracy. To depict the numerical inaccuracy caused by the modelling approach, again dimensionless Bingham formulations are used in Figure 12 and Figure 13 after De Schryver and De Schutter [55]. From these results, it is clear that the lower the pressure number Pn or the lower the discharge number $\hat{Q}$, the relatively less accurate the simulation becomes compared to the Buckingham–Reiner theory.

**Figure 14:**
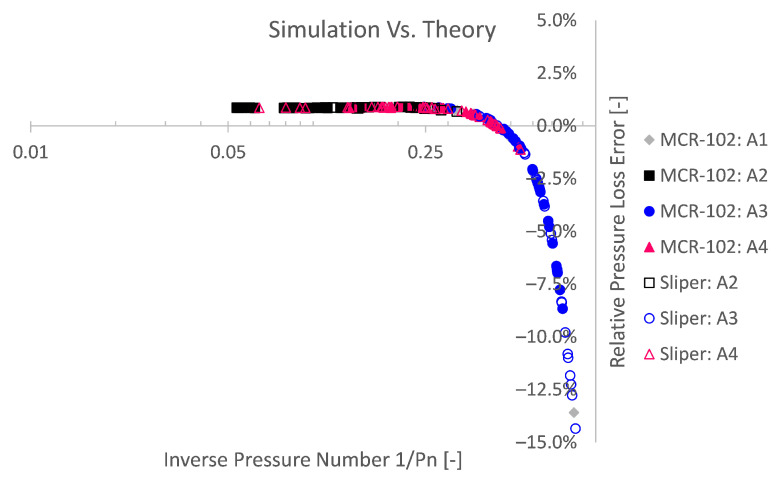
The relative pressure loss error as a function of the inverse pressure number is equivalent to the degree of plug formation. The ratio of plug radius $R_{p}$ to pipe radius *R* equals the inverse pressure number 1/Pn.

Reminiscent to the numerical Bingham stability [25,57,58], one could similarly define a certain flow condition boundary below which a lower relative accuracy could be expected. Although former studies were conducted in the framework of turbulent transition instability, one may also define stability towards the lower Reynolds number end, i.e., laminar unyielded flow, potentially leading to numerical instability. Doing so, based on the considered bi-viscous approach (GNAR=1000), one could state that below a dimensionless discharge $\hat{Q}$ of ca. 0.069 or a pressure number Pn below ca. 1.25 (5/4), less numerically accurate results could be expected. This means that, when the yield stress contribution becomes more than 4/5 (PN≤1.25) of the pressure loss or flow regime, less accurate simulations are expected. Numerical inaccuracy or even instability occur for quiescent flow conditions (i.e., low or zero shear zones) [25,28,29]. When the unyielded region becomes relatively large, less accurate results are obtained due to the (un)regularised viscoplastic problem [25,28,29]. A pressure loss number of below 1.25 (Pn≤5/4) means a plug radius of at least 80% of the pipe radius (i.e., $R_{p}/R=1/Pn\geq 4/5$) in accordance with Figure 14 or 64% in terms of plug area (i.e., $A_{p}/A=1/Pn^{2}\geq 16/25$). This of course is only valid for the considered bi-viscous regularisation approach (GNAR=1000) and pipe flow simulations.

This conclusion is in line with Frigaard and Nouar [25] and Jahromi et al. [28], where the closer the applied stress comes to a fully unyielded flow, the higher the error becomes. These errors can become high, even up to 100%, and hence flow start-up problems using a regularised approach are of questionable nature with regard to accuracy. Therefore, the yielded region and regularisation approach should always be verified in the numerical approach to ascertain proficient numerical outcome.

Nevertheless, dimensionless numbers, such as the Bingham number Bn or more specifically the discharge and pressure number ($\hat{Q}$ and Pn), could serve as an indication of whether numerical inaccuracies or even numerical stability problems could be expected due to the so-called unregularised viscoplastic problem. Concluding the numerical reliability irrespective of its rheological input, based on literature and the Buckingham–Reiner theory, it can be stated that the OpenFOAM simulations are significantly accurate and in significant agreement with the theory. Some rules of thumb have been outlined to ensure adequate numerical accuracy related to the considered regularisation.

To further assess the reliability of the numerical outcome, an assessment was also performed with respect to its rheological input.

## 5. Reliability Dependent on Rheological Input

To assess the numerical reliability of pipe flow simulations (using OpenFOAM) dependent on its rheological input, a comparison was made with the experimental results obtained from a small-scale pumping experiment. As described in the experimental framework section, four mix designs (A1 to A4) were rheologically characterised based on two different devices (MCR-102 and Sliper, apart from A1) and tested in a small-scale pumping circuit. All experimental pumping series were simulated, each based on two different rheological input values. The first rheological input is the characterisation by the MCR-102 rheometer, the second by Sliper. A single discharge value translates to a single simulation. For all rheological values (Table 3) and discharges combined, this resulted in 213 simulations in total.

### Comparison with Experiments

**Figure 15:**
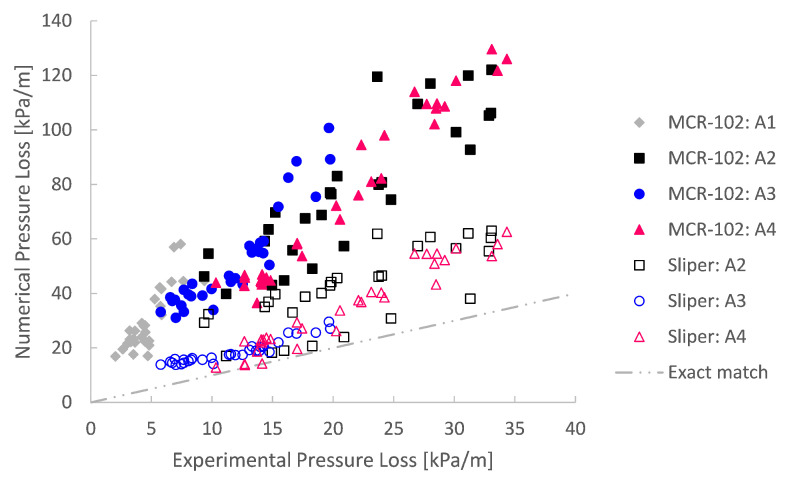
Comparison of numerically simulated pressure loss with experimentally obtained pumping results, for four different mix designs (A1 to A4) and based on rheological input from the MCR-102 rheometer and the Sliper.

For the analysis, the relative pressure losses obtained by the simulations were compared with the experimental ones. Comparing the numerical prediction with the experiment on a one-to-one basis in Figure 15, it is clear that an exact match was rarely the case. The numerical simulations overpredicted the pumping experiment, whether the rheological input was based on the MCR-102 rheometer or the Sliper. It is however remarkable that the rheological input based on Sliper performs better than the results based on the MCR-102. Some Sliper results even lie in the near vicinity of an exact match. The origin and potential influences of this mismatch are discussed in Section 6.

**Figure 16:**
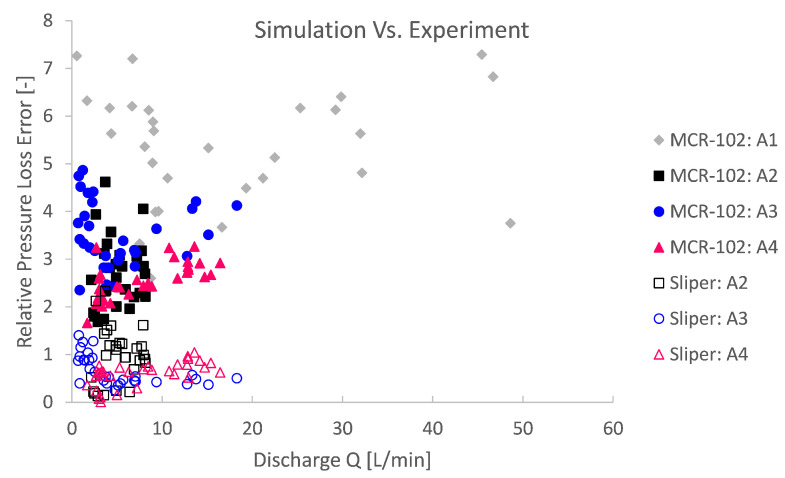
The relative simulation pressure loss error compared to the pumping experiment series indicates that the rheolocigal input from the Sliper is in better agreement than that based on MCR-102.

**Figure 17:**
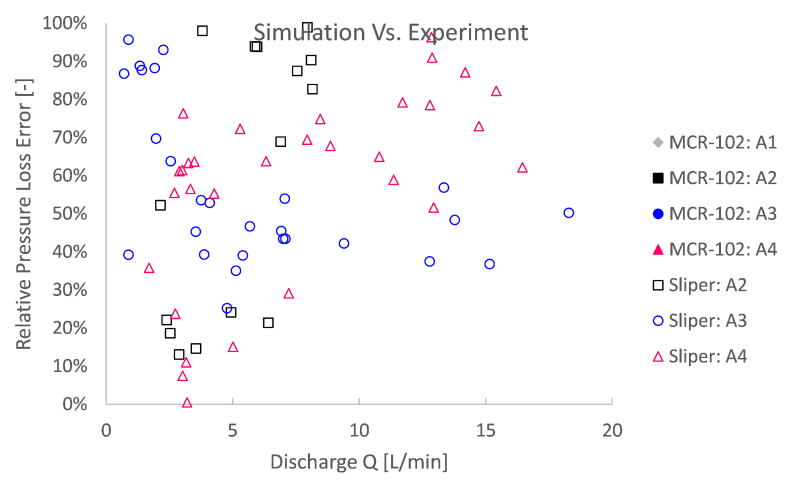
A close-up of the comparison between numerical simulations and pumping experiments shows that the rheological input based on Sliper is significantly better than that for MCR-102, with relative errors ranging from 0% to ca. 100%.

For a quantitative reliability assessment of the pipe flow simulations in comparison with the conducted pumping experiments, again, a relative pressure loss error is computed. The relative error depicted in Figure 16 and Figure 17 is, this time, the absolute difference between the simulation and the pumping experiment and relative to the experiment.

High experimental relative errors were observed. The simulation errors based on MCR-102 were 2 to even 7 times higher than the experiment (Figure 16). For rheological input from the Sliper, the relative error is more limited from 0 to ca. 100% error (Figure 17). This could mean several things. First, this could mean that the rheological characterisation is biased or completely unreliable in a different application or context. Second, this could mean that the theory is biased and that a dominant physical phenomenon was overlooked and, therefore, not simulated. The third explanation is a combination of the former two to some degree: an unreliable rheological bias and physical phenomenon bias. Last, there might be an experimental source of error to an unknown extent.

## 6. Discussion

The general rules of conduct for appropriate regularisation were not explored in this work. As for most works [21,25,28,29], the regularisation itself is ad hoc and applied for a specific application or problem [25]. Restrained to the context of this work, some general rules of thumbs may be defined, for which less accurate numerical results can be expected. These are only valid for an apparent viscosity ratio GNAR=1000, as advised by Bercovier and Engelman [22], and for the specific case of pipe flow of cementitious paste suspensions. A high maximum apparent viscosity ratio up to 1000 can be at the cost of numerical stability. In the work of Syrakos et al. [26], the solver could only cope with simulations with ratios up to 400. Therefore, it is noteworthy to use under-relaxation to a limited extent, as in this work. This is highly beneficial for numerical stability while still allowing for a very strict regularisation parameter. Even though relaxation smears out the simulation to a limited extent, it allows for simulations that were otherwise infeasible.

In line with O’Donovan and Tanner [23], Jahromi et al. [28], Ahmadi and Karimfazli [29], it is concluded that, for flows where the unyielded region is large—or equivalently, a low pressure number Pn and dimensionless discharge $\hat{Q}$—less accurate results are expected. Therefore, similar to that stated by Frigaard and Nouar [25], lubrication layer flows are more of a concern because they convey a very large plug zone. This is especially the case for concrete flows. Even though higher shear rates are expected near the pipe wall and therefore higher local related dimensionless discharges (based on the lubrication layer properties) are expected, the numerical outcome and especially the stability are questionable. To ascertain accuracy for these kind of flows, a regularisation parameter as close as possible to zero (or an apparent viscosity cut-off close to infinity: GNAR→+∞) would be necessary. However, as the regularisation parameter tends towards zero, the simulation tends to blow up [27]. Therefore, an extensive regularisation study on lubrication pipe flows is an important study that could be conducted in the future, especially for the practice of concrete flow simulations.

Despite very accurate results compared to the theory, a high relative experimental error exists, and rarely, an exact match was found between the numerical simulation and experiments. This could be explained by a bias in rheological input, a bias in overlooked underlying physical phenomenon or a combination of both. A fourth error source could be the experimental accuracy or set-up. The pressure sensors have a finite accuracy. Additioanlly, a residual paste membrane found in front of the pressure sensor—but limited to only a few millimetres thickness—could have reduced recorded pressure losses to an unknown extent. Lastly, the accuracy of the density measurement may have had an influence on the discharge computed from the load cell and therefore an indirect influence on the results.

A rheological bias may very well be the case, since significant differences exist in rheological characterisations of cementitious suspensions [14,15]. Hence, the rheological input is certainly a source of uncertainty, as also reported by Schaer et al. [21]. In order to cope with the potentially overlooked rheological bias, it would be more appropriate to obtain rheological input from an application that is closely related to the nature of the aimed engineering application. However, one should be cautious not to overlook any other physical phenomena biases. Doing as such, more reliable numerical predications can be achieved. In fact, one could even consider this to be a model calibration. If for instance rheological data are obtained from a pumping experiment itself, which is then used as the rheological input, it is by definition calibration. This calibration could then be used to predict a new pumping experiment, be it with the same mix design or perhaps even for a different application of the same mix sample.

Despite the uncertain character of rheometers, it does not mean that they cannot be used to assess rheological properties. They still serve as a reference framework within which rheological differences can be addressed. The difficulty lies in a change of context.

A theoretical bias could also lie at the origin of the simulation mismatch. In order not to be biased, it is the researcher’s or engineer’s task to consider the adequacy of the considered numerical or, by extension, the theoretical modelling approach. In the case of simple cementitious paste pipe flows, the behaviour is well established by a Bingham approach and a behavioural bias by, for instance slippage, or a wall lubrication layer is therefore not expected [38,59]. Even though the rheological input based on the Sliper tests was significantly better than for the MCR-102, the results mostly overestimated the pumping experiment. Since the flow inside the Sliper device is not fully developed, the rheological parameters are expected to be at least overestimated, and therefore, it could partially explain the overprediction.

Although no wall lubrication layer is expected in the case of paste, there may have been some influence by the pre-usage water cleaning procedure. Even though all residual water was pumped out, limited intermixed water near the pipe wall surface, slightly altering the mixture composition, may have had a contribution in reduced flow resistance. From a future perspective, it may therefore be important to experimentally investigate the influence of a pre-wetting procedure of pumping pipelines, whether that is using water or cement-paste itself before the application of concrete pumping.

In the case of more complicated pipe flows of fresh concrete, a bias may occur due to not considering the so-called lubrication layer approach. Although no concrete and only cementitious pastes and their modelling were considered in this work, knowing whether such a layer could adequately be simulated is another important interpretation of this work since the lubrication layer is assigned to the formation of a cementitious-like layer of paste attached to the vicinity of the pipeline wall. Over the past decade, this phenomenon has been investigated and even though some promising approaches were established, it is still an ongoing research subject on how to adequately model this so-called lubrication layer phenomenon [34,60,61,62].

From a preliminary projective point of view, one could perhaps extend the reliability interpretation of this work to more complicated flows such as concrete. By making use of rheological input closely related to the pumping application, it could be expected that modelling an additional wall lubrication layer effect would be in significant agreement with related theoretical models, such as the model of Kaplan [63] or modified equations of Kwon et al. [16]. Indeed, in the work of Secrieru et al. [34], such numerical simulations with a lubrication layer have been performed and a good agreement can be observed between the numerical simulation and the analytical equations. Despite a good agreement between their successful simulations, lubricational flows in general, however, form a concern from the viewpoint of numerical accuracy and stability, similar to that in Frigaard and Nouar [25]. As mentioned before, an elaborate regularisation accuracy study for lubrication layer pipe flows would especially facilitate engineering practices to simulate concrete pumping. Aside from that, the fact that their numerical model was calibrated for the lubrication layer thickness based on the Sliper rheometry, which lies more close to the nature of concrete pumping itself, forms additional evidence that more reliable simulation results can be obtained by rheological parameters obtained from rheometry configurations as close as possible to the aimed application.

Despite such a preliminary interpretation and from a future perspective, it would be relevant to assess the numerical reliability including a lubrication layer approach. Since not only cementitious paste but also concrete flow simulations are important in construction processes, where the wall lubrication layer effect may not be overlooked. Again, the reliability could be assessed from both a theoretical point of view as an experimental viewpoint. Since no closed analytical expressions are available for a modelling approach by particle migration, the reliability could be assessed for a dual Bingham fluid approach. Closed analytical Poiseuille flow extensions are namely available, such as the model of Kaplan [63] or modified equations of Kwon et al. [16]. In the meantime, the influence of numerical regularisation could be investigated for such flows.

## 7. Conclusions

To build confidence in and to answer the question on numerical reliability, simplified numerical simulations of horizontal, laminar Bingham pipe flow were assessed for their outcome. Specific to flow simulations of cementitious Bingham paste suspensions, and by extension to model fresh concrete construction processes for engineers in practice, the assessment was performed irrespective of and dependent on their rheological input.

The reliability assessment irrespective of the rheological input revealed that numerical finite volume simulations by OpenFOAM are very accurate compared to the theoretical equations. From a practical, construction process engineering viewpoint, an accuracy of 1% is reasonable. At least if the simulations are performed by meeting the defined rules of thumb: a proper regularisation approach (e.g., a bi-viscous approach with $GNAR=\mu_{max}/\mu \approx 1000$) and for higher discharge regimes defined by the dimensionless pressure number (Pn>1.25) and/or discharge ($\hat{Q}>0.069$). More accurate results can be obtained if a higher mesh resolution is considered and if higher order numerical schemes are considered. Although it should be limited, artificial under-relaxation appeared to be a promising technique to cope with potentially occurring numerical instabilities due to the (un)regularised viscoplastic problem [25,43]. This is especially important for lubrication flows, such as concrete pumping, although accuracy and stability are still of concern for lubrication type flows.

Based on the reliability dependent on the rheological input, it was shown that, due to the uncertain character of rheological input data, more reliable numerical predictions can be obtained by considering rheometry that lies more close to the configuration of the engineer’s aimed application. In that way, one is able to cope with a possible bias due to rheological uncertainty. One should however be cautious not to overlook a physical phenomenon. All significant physical phenomena should be modelled as well. Unlike cementitious pastes, this is, for instance, the case for concrete pumping where the wall lubrication layer effect cannot be overlooked.

Even though the numerical simulations used in this work may not cover the full spectrum of numerical simulations for the behaviour of cementitious paste suspensions, it still forms a certain benchmark for a CFD finite volume modelling approach in OpenFOAM to model cementitious paste suspensions as a Bingham fluid important for the construction industry.

From a future perspective, it would be especially of interest for engineering practice to build confidence in CFD simulations by performing a similar reliability assessment considering an additional lubrication layer effect at the pipe wall. One could similarly compare the numerical outcome irrespective of the rheological input with related theories as well as make an assessment based on rheological input by comparing it with experiments. Simultaneously, with regard to accuracy, one may also address the concern of regularisation and stability for lubrication layer flows. Another important study that could be of experimental interest is the influence of a pre-wetting or pre-lubricating procedure for concrete pumping.

## Figures and Tables

**Table 1:** Pipe flow boundary conditions of the velocity field *U* and the pressure field *p*. Herein, *Q* is the imposed discharge, uniformly distributed over the inlet surface *A*.

Boundary	Field	Type	Definition	
Inlet	*U*	Dirichlet	uniform value	U=Q/A
	*p*	Neumann	zero gradient	∇p=0
Wall	*U*	Dirichlet	noSlip	U=0
	*p*	Neumann	zero gradient	∇p=0
Outlet	*U*	Neumann	zero gradient	∇U=0
	*p*	Dirichlet	zero value	p=0

**Table 2:** Conceptual overview of rheological mixture designs of the considered four Bingham model pastes (A1 to A4), as a function of the water (W), powder (P) and superplasticiser (SP) content.

Mix Designs	Plastic Viscosity μ				
	Low		High		
Yield Stress $\tau_{0}$	Mix	W/P	Mix	W/P	SP/P
**Low**	A1	0.40	A2	0.20	0.175%
**High**	A3	0.33	A4	0.25	0.100%

**Table 3:** Rheological overview of several mixture design samples, depicting the paste density ρ, Bingham yield stress $\tau_{0}$ and plastic viscosity μ obtained from different rheometers.

		MCR-102		Sliper	
**Mixture**	ρ	$\tau_{0}$	μ	$\tau_{0}$	μ
**Sample**	**[kg/L]**	**[Pa]**	**[Pa.s]**	**[Pa]**	**[Pa.s]**
A1-1	1.776	55.5	0.5	N/A	N/A
A1-2	1.786	69.7	0.7	N/A	N/A
A1-3	1.792	67.7	0.7	N/A	N/A
A1-4	1.794	94.1	0.9	N/A	N/A
A2-1	2.035	57.2	8.5	44.3	4.6
A2-2	2.075	65.7	9.1	32.2	3.6
A2-3	2.047	43.6	10.3	62.8	4.6
A3-1	1.903	150.5	2.6	73.4	0.6
A3-2	1.865	165.6	2.7	76.3	0.6
A3-3	1.896	181.1	2.8	79.0	0.5
A4-1	1.980	135.1	4.6	23.0	2.2
A4-2	1.979	129.3	4.8	67.3	2.3
A4-3	1.981	110.7	5.0	65.6	2.2

## Data Availability

The data presented in this study are available on reasonable request from the corresponding author.

## Appendix A. Simulation Settings

An overview of the numerical discretisation schemes used is depicted in Table A1. The numerical solver and respective settings are summarised in Table A2.

**Table A1:** Discretisation scheme settings in OpenFOAM.

Discretisation	Field	Scheme
ddtSchemes	default	Gauss linear
gradSchemes	default	Gauss linear
	grad(p)	leastSquares
divSchemes	default	none
	div(phi,U)	Gauss linear corrected
laplacianSchemes	default	none
	laplacian(nu,U)	Gauss linear corrected
	laplacian(1|A(U),p)	Gauss linear corrected
interpolationSchemes	default	linear
snGradSchemes	default	corrected

**Table A2:** Solver solution settings in OpenFOAM.

Application	nonNewtonianIcoFoam	
Pressure-Velocity Coupling	PISO	
nCorrectors	5	
nNonOrthogonalCorrectors	0	
	**Fields**	
**Solver Settings**	*p*	*U*
Solver	GAMG	smoothSolver
Smoother	GaussSeidel	symGaussSeidel
Tolerance	1 × 10^−6^	1 × 10^−6^
Relative Tolerance	0.1	0
Final Relative Tolerance	0	
Relaxation Factor	1.0	0.95
